# Surgical Approaches for Possible Positions of an Olfactory Implant to Stimulate the Olfactory Bulb

**DOI:** 10.1159/000529563

**Published:** 2023-03-30

**Authors:** Susanne Menzel, Iordanis Konstantinidis, Marco Valentini, Paolo Battaglia, Mario Turri-Zanoni, Giorgio Sileo, Giulia Monti, Paolo Giocondo Maria Castelnuovo, Thomas Hummel, Alberto Macchi

**Affiliations:** ^a^Smell and Taste Clinic, Department of Otorhinolaryngology, University Hospital Carl Gustav Carus, Technische Universität Dresden, Dresden, Germany; ^b^Smell and Taste Clinic, 2nd ORL Academic Department, Aristotle University, Thessaloniki, Greece; ^c^Division of Otorhinolaryngology, Department of Biotechnology and Life Sciences, University of Insubria, Varese, Italy

**Keywords:** Olfactory dysfunction, Olfactory implant, Sinus surgery, Smell, Surgery

## Abstract

**Introduction:**

Current scientific developments seem to allow for an “olfactory implant” in analogy to cochlear implants. However, the position and surgical approaches for electrical stimulation of the olfactory system are unclear.

**Methods:**

In a human anatomic cadaver study, we investigated different endoscopic approaches to electrically stimulate the olfactory bulb (OB) based on the following considerations: (1) the stimulating electrode should be close to the OB. (2) The surgical procedure should be as non-invasive and safe as possible and (3) as easy as possible for an experienced ENT surgeon.

**Results:**

In summary, the endoscopic intracranial positioning of the electrode via a widened ostium of the fila olfactoria or a frontal sinus surgery like a Draf IIb procedure is a good option in terms of patients' risk, degree of difficulty for ENT surgeons, and position to the OB. Endoscopic intranasal positioning appeared to be the best option in terms of patient risk and the degree of difficulty for ENT surgeons. Although a bigger approach to the OB using a drill and the combined intranasal endoscopic and external approach enabled a close placement of the electrode to the OB, they do not seem relevant in practice due to their higher invasiveness.

**Conclusion:**

The study suggested that an intranasal positioning of a stimulating electrode is possible, with placements beneath the cribriform plate, extra- or intracranially, applying elegant surgical techniques with low or medium risk to the patient and a close placement to OB.

## Introduction

Approximately 20% of the population has an olfactory impairment, with about 5% being functionally anosmic [1, 2, 3]. The reasons for this are manifold, but age-related olfactory impairment is the most frequent cause and is of great importance, especially due to demographic developments [2]. Other main causes of olfactory impairment are sinonasal disorder, viral infections, or traumatic brain injury [4, 5], whereas recently the cases of postviral olfactory loss are increasing due to the ongoing COVID-19 pandemic [6]. The loss of smell has an impact on the quality of life. Patients with olfactory dysfunction are more depressed, have more accidents, experience changes in their sexuality, and have no flavor perception during eating and drinking [7].

Currently available treatments are, e.g., olfactory training, medication, and surgical approaches that exhibit limitations [8, 9]. Nevertheless, depending on the cause, the prognosis for olfactory loss is often poor. The improvement for long-standing postviral olfactory dysfunction is about 32% within 13.7 ± 0.8 months [10]. For posttraumatic olfactory dysfunction, the results were even worse, showing an improvement of only around 10% within 13.4 ± 0.9 months. Similar studies also reported a better outcome for postviral cases compared to posttraumatic ones, with an improvement of 36% versus 25% within 15 months [11]. The recovery rate is depending on the duration of the disease, patient age, remaining olfactory function, and probably the volume of the olfactory bulb (OB) [10, 11]. Still, there are a large number of patients with impaired olfactory function, who do not respond to any treatment. Existing treatment options should therefore be amplified [12]. The fundamental technology of an olfactory implant exists, consisting of sensors that detect and differentiate odors [13, 14]. The direct stimulation of the olfactory system relayed from these sensors could be an option to bridge peripheral damage to the olfactory epithelium or fila olfactoria [15]. However, it is still unclear where stimulation should be performed to get the desired sensory effect [12, 15, 16].

Further, the potential placement of a sensor should be discussed. If the sensor is placed extra-nasally, the contextual perception of olfactory stimuli would be missed due to missed process of retronasal smelling while eating. By moving the soft palate when swallowing, the air is pumped into the olfactory cleft so that the perception of aromas can take place. The aspect of retronasal smelling while eating is a strong argument for the intranasal location of a possible sensor of an olfactory implant. Furthermore, sniffing itself has an important role in olfaction [17]. On the level of electrophysiological processing of odors, it has been shown that there are theta frequency bands in the piriform cortex with unique time courses relative to sniff onset measured with intracranial EEG. That could be comparable to a set alarm with every breath we take waiting for an odorous stimulation [18]. Primary and secondary olfactory cortices are activated in response to odorless sniffs, i.e., the piriform cortex of the temporal lobe, and the medial and posterior orbitofrontal gyri of the frontal lobe [19]. In a small group of subjects of anosmic patients, it was shown that sniffing activated similar brain areas in the fMRI as in healthy subjects [20]. Previous studies also have shown that congenital anosmia is not characterized by atypical functional connectivity in the olfactory cortex and functional connectivity within the predefined olfactory network. This could be interpreted as a certain resting state which is in contrast to the changes in the visual cortex in blindness. It also may suggest that the olfactory cortex does not require early sensory input [21]. This appears to be a good basis for the cerebral processing of activation in the olfactory system, which could be initiated by an olfactory implant, even when the respective individuals did not smell for a long time.

Electrical stimulation has first been tried in the 19th century [22]. On the one hand, some unpleasant perceptions, such as something burnt, were described [22, 23]. On the other hand, there are a few studies describing different olfactory perceptions. In children with medically resistant epilepsy, stimulation via subdural electrodes implanted in the frontal lobe was done [24]. Out of 16 patients, 11 described an olfactory sensation. While 9 patients had an unpleasant smell, two perceived a pleasant smell (like strawberries or good food). In an additional study, 3 out of 5 patients who underwent sinonasal surgery reported reproducible olfactory sensations (onion-like, fruity and bad, antiseptic-like and sour) after electrical stimulation of the lateral lamella of the cribriform plate [25]. Hence, it seems to be possible to produce olfactory sensations through electrical stimulation in different areas. The authors suggested electrical stimulation at the level of OB could be a proof of concept to develop an olfactory implant. The OB is of particular importance in the processing of olfactory stimuli. A study on rats showed that different spatial patterns of neural activity were obtained for different odors. Furthermore, direct stimulation of the OB at different locations generated different spatial patterns of neural activity [26]. This could recreate normal olfactory perception which is based on pattern analyses because every odor produces a different pattern of activation [27]. A study on rodents has shown that electrical stimulation via a cochlear implant placed in a deafferented OB evoked localized field potential responses [28]. Moreover, the OB is probably easier to access than other structures of the olfactory system. Elegant approaches to the OB are offered by endoscopic sinonasal surgery to avoid frontal craniotomy [29, 30]. There are different approaches to reach the OB: a supraorbital keyhole approach via an eyebrow incision [31], and a combined transnasal and transfacial approach, which is done in olfactory neuroblastoma [30]. In a previous study, the approach to OB was studied using a midline olfactory implant with a transseptal approach and two types of electrode array placement: “extracranial under the cribriform plate and extradural between the OB after transcribriform removal of posterior two-thirds of crista galli” [32]. The authors mentioned the risk of cerebrospinal fluid (CSF) leak for the extradural approach. To date, there is no olfactory implant or electrode certified for eliciting olfactory percepts, through implantation in the OB. The study was therefore performed on existing electrodes used for cochlear implants and with a type of wire used for intracranial stimulation.

In this pilot study, we investigated different operative approaches in endoscopic surgery to stimulate the OB, which seems to be promising concerning surgical accessibility and the influenceability of olfactory perceptions by stimulation. The basic assumption for the approach was that the OB should be stimulated electrically. Hence, the following principal considerations were used: (1) the stimulating electrode should be close to the OB, because of the distribution of the electrical field energy. (2) The surgical procedure should be as non-invasive and safe as possible for the patient. (3) Furthermore, the procedure should be as easy as possible by an experienced ENT surgeon.

## Materials and Methods

In a human anatomic cadaver study on two heads of fresh human cadavers, a total of 6 different options of approaches were performed within the framework of endoscopic sinonasal surgery by a team of ENT surgeons experienced in skull base surgery. Therefore, similar tissue properties existed in the living, so that the surgical aspect of the access can be studied as accurately as possible. The position of the electrode is evaluated concerning the following parameters: patient risk, degree of difficulty for an experienced surgeon, and electrode placement. The patient risk was determined based on the invasiveness of the approach, with particular consideration given to CSF leaks and anatomically proximal structures that might potentially be damaged. All approaches that cross the base of the skull have a risk of CSF leakage and ascending infection such as meningitis, although this depends on the invasiveness. The degree of difficulty for experienced surgeons was evaluated by three surgeons who were performing the surgery. After the implantation of the electrode, the position was controlled using a CT scan and for 2 options using intracranial dissection to analyze the placement of the electrode. Radiopaque wires of a different rigidity (outer diameter <1 mm, length 50–80 mm) and a demonstration specimen of a cochlear implant (Cochlear, Sydney, Australia) were used as models of an OB stimulation electrode.

**Fig. 1:**
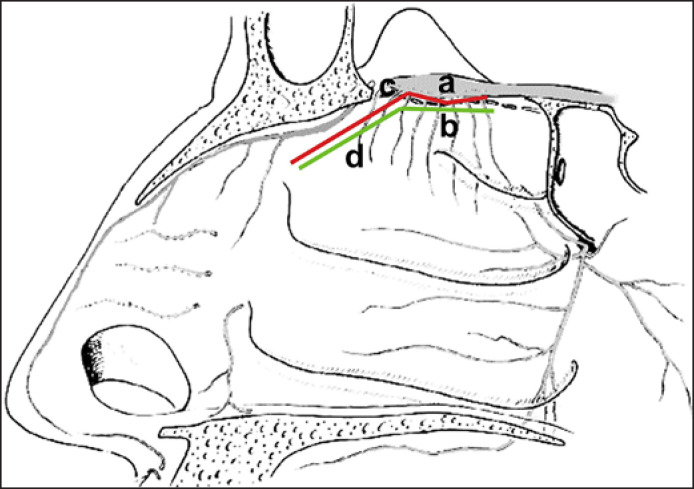
Schematic representation of the electrical stimulation options of OB. **a**, OB; **b**, fila olfactoria; **c**, red line intracranial position of the stimulator of the OB; **d**, green line, intranasal position of a stimulator from the inside of the nasal cavity.

**Fig. 2:**
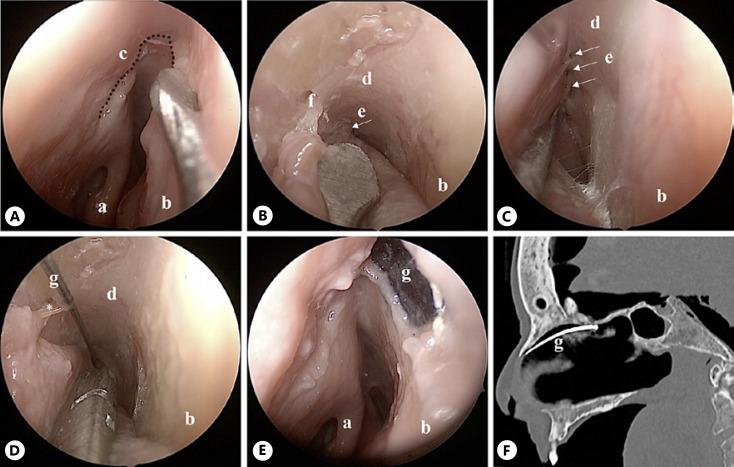
Endoscopic intranasal positioning of the electrode (approach 1), right nasal fossa. **A** Harvesting of the U-shaped mucosal flap. **B, C** Preparing the olfactory cleft as posterior as possible. **D** The electrode is placed in the olfactory cleft. **E** The mucosal flap is replaced to cover the electrode so that only a sensor attached to the electrode is seen on endonasal mucosa. **F** Sagittal CT scan showing a part of the intranasal positioning of the electrode. **a**, middle turbinate; **b**, nasal septum; **c**, dotted line, mucosal incision for the U-shaped flap; **d**, olfactory cleft; **e**, fila olfactoria; **f**, septal branch of anterior ethmoidal artery; **g**, electrode.

**Fig. 3:**
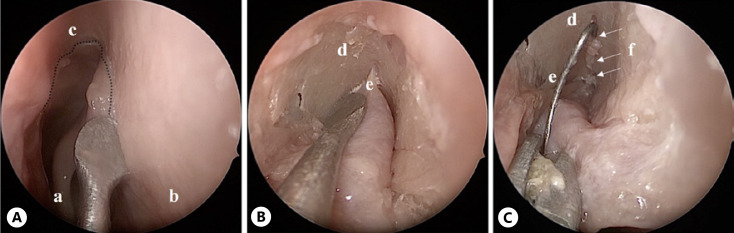
Endoscopic intracranial approach through the ostium of the first olfactory fila (approach 2), left nasal fossa. **A** Harvesting of the U-shaped mucosal flap. **B** Preparing the olfactory cleft. **C** The electrode is placed inside the ostium of the first olfactory fila. **a**, middle turbinate; **b**, nasal septum; **c**, mucosal incision for the U-shaped flap; **d**, olfactory cleft; **e**, vascular branch; **f**, fila olfactoria; **e**, electrode.

Based on the anatomical structures, there are three ways of stimulating the OB: (1) endoscopic intranasal position of a stimulator in contact with the skull base (shown in Fig. 1d), (2) endoscopic intracranial position of the stimulation under the OB with different accesses (shown in Fig. 1c), or (3) combined (endoscopic and external) positioning of the stimulator under the OB. The study was conducted according to the Declaration of Helsinki and had been approved by the local ethics board (Insubria Board of Ethics, approval number 0033025/2015). Body donors provided written informed consent.

## Results

### Endoscopic Intranasal Positioning of the Electrode

(1) An U-shaped mucosal flap (shown in Fig. 2A, c) was used starting at the level of the axilla of the middle turbinate to get access to the skull base and hereby to the olfactory cleft. Followed by the preparation of the olfactory cleft and its penetrating fila olfactoria as a tunnel as posterior as possible (shown in Fig. 2B, C), see online supplementary material Video 1 (for all online suppl. material, see www.karger.com/doi/10.1159/000529563). Care must be taken when manipulating close to vascular branches (shown in Fig. 2B, f). The electrode was placed directly underneath the cribriform plate in contact with the skull base, and the mucosa flap was replaced (shown in Fig. 2D, E).

A major advantage of this surgery is that it is less invasive for the patient and relatively easy and quick (15 min) for the experienced surgeon to perform. The surgical positioning of the electrode at the olfactory cleft is well feasible after preparing the mucosa. With the electrode placed in the olfactory cleft, it has a close topographical relation to the OB. Unfortunately, this is not visible clearly due to the 2D representation (shown in Fig. 2F, g). A disadvantage is the relatively long distance of the electrode from the OB due to the skull base between them, so stimulation may be difficult depending on the anatomic position.

### Endoscopic Intracranial Positioning of the Electrode

To study the endoscopic intracranial approach, different accesses have been performed.

**Fig. 4:**
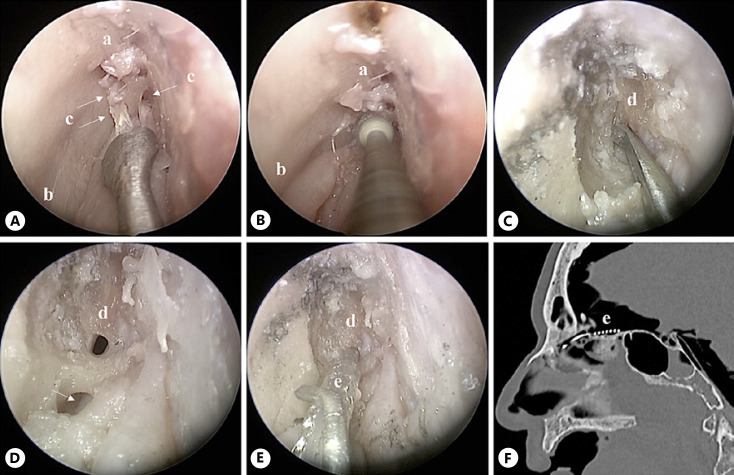
Endoscopic intracranial approach through the cribriform plate (approach 3), right nasal fossa. **A** The olfactory cleft is prepared; olfactory fila are cut. **B, C** The cribriform plate is drilled until the dura is reached. **D** A small hole in the dura is made. **E** The electrode is inserted inside the hole. **F** Sagittal CT scan showing the intracranial positioning of the electrode close to the OB. **a**, olfactory cleft; **b**, nasal septum; **c**, arrows, olfactory fila; **d**, dura; **e**, electrode.

**Fig. 5:**
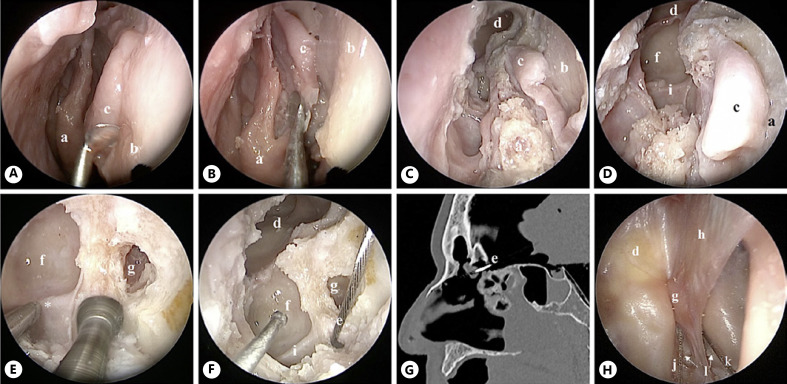
Endoscopic intracranial approach through Draf IIb procedure (approach 4), right nasal fossa. **A** Septal flap is harvested and positioned safely medially to the middle turbinate (**B**). **C** Draf IIb is performed and first fovea ethmoidalis with AEA are exposed (**D**). **E** Drilling of the cribriform plate, medially to the cranial insertion of the common basal lamella and posteriorly to the AEA, until the dura is exposed. **F** Positioning of the electrode; which will be covered with the septal flap afterward. **G** Sagittal CT scan showing the intracranial positioning of the electrode. **H** View from above (a craniotomy was performed with endoscopic assistance) showing the transillumination on the posterior wall of the left frontal sinus and the intracranial placement of the electrodes of approaches 3 and 4. **a**, middle turbinate; **b**, nasal septum; **c**, septal flap; **d**, frontal sinus; **e**, electrode; **f**, first fovea ethmoidalis; **g**, crista galli; **h**, falx cerebri; **i**, anterior ethmoidal artery; **j**, the electrode of approach 3; **k**, electrode of approach 4; **l**, cribriform plate.

**Fig. 6:**
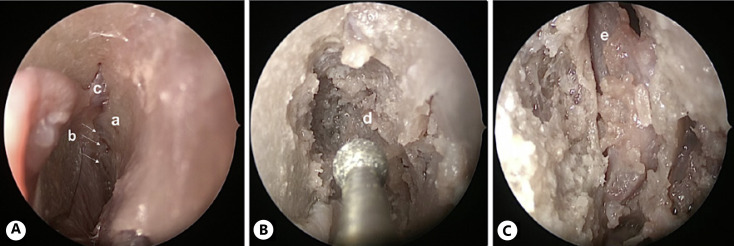
Endoscopic intracranial approach through the preparation of olfactory cleft (approach 5), left nasal fossa. **A** The olfactory cleft is prepared; showing olfactory fila and septal branch of anterior ethmoidal artery. **B** The cribriform plate is drilled until the dura is reached. **C** Intracranial approach, showing OB. **a**, olfactory cleft; **b**, arrows, olfactory fila; **c**, septal branch of anterior ethmoidal artery; **d**, dura; **e**, OB.

**Fig. 7:**
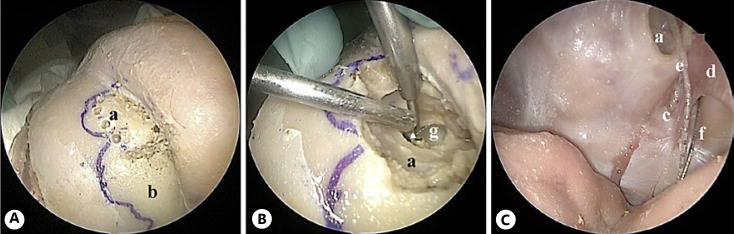
Combined approach through a frontal osteoplastic flap (approach 6). **A** The borders of the frontal sinuses are marked in blue on the anterior wall, the left frontal osteoplastic flap is harvested. **B** Drilling of the frontal sinus posterior wall. **C** The electrode is inserted through the window created on the frontal sinus posterior wall. **a**, left frontal sinus; **b**, right frontal sinus; **c**, cribriform plate; **d**, falx cerebri; **e**, electrode of approach 5; **f**, electrode of approach 4; **g**, drilled out posterior wall of left frontal sinus.

(2) After the U-shaped mucosal flap was harvested (shown in Fig. 3A) and the olfactory cleft was visualized, the first olfactory filament was cut (shown in Fig. 3B), and an electrode was inserted through the first ostium of the fila olfactoria in the cribriform plate (shown in Fig. 3C). This procedure was relatively easy to implement. Due to the anatomical location of the OB posteriorly of the first olfactory fila, it was relatively far from the OB. If a rigid electrode is used, there is a potential risk of electrode mal-positioning above the bulb toward the brain. When a more flexible electrode was used, the structure of the fila olfactoria seemed complicated to push the electrode forward underneath the OB.

(3) A more posterior ostium of the fila olfactoria in the cribriform plate was widened using a diamond straight shaft drill (diameter 1.8 mm) (shown in Fig. 4B), which is also used in ear and sinus surgery. Through the new ostium, the electrode was inserted (shown in Fig. 4E). The position of the electrode was intracranial, close to the OB (shown in Fig. 4F, e). After performing a craniotomy with endoscopic assistance, a close position to OB was shown (shown in Fig. 5H, j). The intra- and perioperative risks are considered moderate due to the intracranial access. In addition, the possibility of a CSF leak is reduced due to the limited skull base opening.

(4) After harvesting a septal flap vascularized by the AEA septal branches, the electrode was placed intracranially performing a Draf IIb procedure [33], which is the resection of the frontal sinus floor between the nasal septum and the lamina papyracea [33, 34]. This access was chosen to provide a better overview of the anatomical structures: frontal sinus, first fovea ethmoidalis, AEA, common basal lamella, and cribriform plate. Afterward, the cribriform plate was drilled (shown in Fig. 5H, l), using a diamond straight shaft drill (diameter 1.8 mm), medially to the cranial insertion of the common basal lamella and immediately posteriorly to the AEA. At the end of the procedure, the septal flap was used to cover the electrode and exposed bone. In the CT image as well as after performing a craniotomy with endoscopic assistance, a close position to OB was achieved (shown in Fig. 5G, e, k).

(5) After preparation of the mucosal flap (shown in Fig. 6A), drilling of a wider area in the olfactory cleft was performed (shown in Fig. 6B) to access OB (shown in Fig. 6C, e). This more invasive strategy crossing the skull base implied a higher risk for the patient, provoked CSF leakage, and the need for skull base reconstruction by definition. Olfactory filaments should be cut to get access to the skull base.

(6) A combined approach with an endonasal and frontal osteoplastic flap approach has been performed: after a bicoronal incision, a frontal osteoplastic flap was harvested, hence entering the frontal sinus with partly endoscopic assistance as a mainly external approach; the posterior wall was then drilled out (shown in Fig. 7B, g) to get access toward the OB (shown in Fig. 7C). This approach poses an additional risk to the patient compared to the other endoscopic intracranial approaches, due to the intracranial access route from the outside. From a surgical point of view, this procedure is time-consuming and somewhat complicated to manage, but it provides a good overview of the surgical area from above. However, it still presents difficulties in the manipulations and positioning of the electrode working from a level above the OB. For an overview of the approaches, see Table 1.

## Conclusion

The general idea of this pilot study in the sense of a human anatomic cadaver study was to identify different approaches and positions of electrodes of an olfactory implant that could potentially stimulate the OB. The present study clearly showed that an intranasal positioning of stimulating electrodes is possible, with placements beneath or above the cribriform plate, extra- or intracranially, applying elegant surgical techniques with low or medium risk to the patient.

The general risk of the endoscopic intracranial positions is the possibility of a CSF leak and local or ascending infections like meningitis, increasing with more complex surgery. These risks depend on the invasiveness of the procedure and individual anatomy. For approach 1 investigated here, the skull base would not be crossed due to the purely intranasal electrode position, so the above-mentioned risks of CSF leak are not taken into consideration. Further, the risk for ascending infections is low. For the other approaches (2–6), the skull base is crossed, so that the above-mentioned risks may occur, whereby the probability of occurrence depends on the invasiveness of the operations. While approaches 2–4 require only a small punctuated opening of the skull base, as this is limited to the diameter of the electrode, in approaches 5 and 6 there is a larger defect of the skull base to get a better anatomical overview. Depending on the surgical damage to the skull base, different reconstructions of it are required. While larger defects of the skull base require complex reconstruction of these, e.g., using a vascularized flap, in some circumstances smaller defects could use fat among other materials such as foils for reconstruction. Furthermore, with an intracranial position, the complexity of the surgery is increasing, so an interdisciplinary treatment with otolaryngologists and neurosurgeons should be considered.

These risks could be reduced by using precise preoperative imaging including 3D reconstruction, the use of intraoperative neuromonitoring, and navigation-guided surgery. In addition, intraoperative imaging should be performed to check the position of the electrode, which is also used in some cases during the placement of cochlear implants [35]. Furthermore, postoperative imaging could be performed to rule out intracranial complications such as hemorrhage in some patients with a higher risk of intracranial complications [36]. To monitor the risk of persisting CSF leakage after reconstruction of the skull base, a preoperative intradural dye injection could be considered. For all surgeries and especially for intracranial ones, there is a risk of wound infections that could lead to encephalitis or meningitis. For all intracranial approaches, we would recommend peri- and postoperative antibiotic therapy.

Due to the less invasive procedure in approach 1, the degree of difficulty for an experienced surgeon is lower compared to the other approaches. All intracranial approaches have a higher degree of difficulty for an experienced surgeon. Approach 4 was a bit more complex from a surgical point of view because of the performed Draf IIb procedure, but it allowed a good overview of the surgical area giving more space for the manipulation of surgical instruments. Approaches 5 and 6 require a reconstruction of the skull base so that the degree of difficulty for a surgeon is high.

For some approaches, additional instruments could be useful. Probably, a more rigid electrode could facilitate the placement of the electrode in approach 1. For approaches 2 and 3, an inserting instrument and a more flexible electrode would be useful for the surgeon. For approach 3, the diode laser could be used to create a small hole in the exposed dura, as in stapes surgery. For all approaches, a nasal packing pushing the mucosal flap upward in the olfactory cleft may be needed for a few days. Especially for approach 1, the nasal packing could be necessary to keep the electrode in contact with the skull base for a few days until the tissue and the electrode stay in close contact. Further, the fixation of the electrode in close contact with the skull base could be difficult and susceptible to postoperative changes in the position.

The electrode got closer to the OB performing the intracranial approaches (2–6) than performing the intranasal approach (1). For approach 1, it might be a problem to stimulate the OB because of the bony structure and tissue between the OB and the electrode. However, the evaluation of the location of the electrode to the OB is more subjective by experienced surgeons. Due to the post-mortem changes of the intracranial structures, it was not possible to measure the exact location of the electrode to the OB. To achieve higher evidence of the statements about the position of the electrode to the target structure in the approaches, precise measurements are required in further studies. Further, it might be necessary to calculate the distribution of the electrical field emanating from the electrode to predict the density of the electrical field at the level of the OB. This could be crucial to allow different patterns for more specific stimulation of various areas of the OB. In addition, extracranial stimulation of the OB could also be considered, as would be possible with electrodes attached to a frame of glasses, for example. An external arrangement of electrodes [37] induced an electrical field with a maximum at the olfactory mucosa and/or the OB.

Based on the pilot study, the endoscopic intranasal positioning of the electrode (approach 1) appears to be the best option in terms of patient risk and the degree of difficulty for experienced ENT surgeons, followed by endoscopic intracranial positioning of the electrode via the natural (approach 2) or widened (using a drill) ostium of the fila olfactoria (approach 3) or via a frontal sinus surgery like a Draf IIB operation (approach 4). Regarding the close placement of the electrode to the OB, the endoscopic intracranial positioning of the electrode seemed to be the best: through the widened ostium of fila olfactoria (approach 3), via a frontal sinus surgery like a Draf IIb procedure (approach 4), via a bigger approach to the OB using a drill (approach 5), and the combined approach (approach 6). Due to the higher invasiveness, approaches 5 and 6 may not seem to be very relevant in practice.

In a previous work, Benkhatar et al. [32] studied on cadavers two possible positions of midline olfactory implants: extracranial under the cribriform plate and extradural between the OB after transcribriform removal of the posterior two-thirds of crista galli. The latter approach required specific bone resection with a high-speed drill. The olfactory implant was placed in the frontal midline with the cable running in a loop through the nasion incision. The authors mentioned a high rate of septal mucosa perforation for the intranasal approach, which could lead to an infection and damage to septal cartilage due to decreased blood supply as a result of mucoperichondrial damage. In our study using the U-shaped mucosa flap (approaches 1–3, 5, 6), we did not do any preparation close to the septal cartilage so that this access has a lower risk in terms of possible damage to septal cartilage. However, the damage resp. removal of the septal cartilage does not necessarily have negative effects and is done intentionally, e.g., in septoplasty. Approach 1 of our study seems to be comparable to the extracranial position of the electrode underneath the cribriform plate in terms of electrode positing, with differences in surgical procedure and possible damage to septal mucosa. For the positioning of the electrode after transcribriform removal of the posterior two-thirds of crista galli, the following adverse effects were observed CSF leakage for all cases, frontal sinus floor penetration, and dural perforation. In our study, there is a risk of CSF leakage for approaches 2–6, with a smaller probability in approaches 2–4 due to a punctuated opening of the skull base and a higher probability in approaches 5 and 6 due to a larger defect of the skull base. In further studies, the quantity of CSF leakage should be addressed, e.g., by using fluorescein-dyed saline perfusion. Currently, the electrode positioning of the previous study and our study cannot be compared with each other using validated measurements because, among other things, postoperative imaging in Benkhatar et al. [32] is lacking.

Different surgical procedures could be used for different degrees of olfactory loss. The intranasal approach (1) could probably be used in hyposmic patients, because of no additional damage to olfactory structures. In the other approaches, it would be necessary to include anosmic patients due to the additional damage to olfactory structures like cutting the fila olfactoria. Regarding the main diagnosis, it might be more promising in patients with hyposmia than in anosmia, because in anosmia it is known that the OB decreases with the duration of the disorder [38]. Furthermore, the shape and the size of the OB are associated with the different olfactory functions [39]. Surveys suggested that 32% of patients with olfactory dysfunction would be interested in an olfactory implant [40]. The decision for an olfactory implant would be based on numerous considerations: patients must be fit for anesthesia and surgery. After the operation, the patient should probably undergo smell training to adapt to the processing of the new activations. Beyond these considerations from the patient's view, the therapy will be costly. In addition, more research is needed in terms of the development of sensors and stimulators.

In the future, an olfactory implant could be a ground-breaking treatment option for selected cases concerning the cause and duration of the disease as well as the age and comorbidities. We acknowledge that this pre-study had some limitations. Improvements to be implemented in future studies would include the following: (1) the exact position of the electrode to the OB should also be determined, e.g., by 3D reconstruction or by using a different tissue fixation to measure the contact of the electrode to the OB. In fresh cadavers, changes of brain structures in the sense of lifting the brain from the skull base were observed. (2) The CSF leakage should be examined in more detail for the different approaches in further studies using colored intracranial fluid. (3) The problem of energy supply has not yet been solved in our proposal. One option would be wireless charging, as is possible with certain mobile devices [41]. This could be done while the user is asleep. A less visible placement of a battery, e.g., mastoidal, would also be conceivable. Further cosmetic aspects were not taken into account yet due to the lack of a complete implant.

In summary, the endoscopic intracranial positioning of the electrode via a widened ostium of the fila olfactoria or a frontal sinus surgery like a Draf IIb procedure is a good option in terms of patient risk, degree of difficulty for ENT surgeons, and position to the OB. Endoscopic intranasal positioning appeared to be a good option in terms of patient risk and the degree of difficulty for ENT surgeons. The results of this study provide a basis for further studies on a larger group of subjects for a more detailed analysis of the feasibility of the approaches, especially approaches 1, 3, and 4. Further studies are needed to identify where to stimulate to get an olfactory percept.

## Statement of Ethics

The study was conducted according to the Declaration of Helsinki and had been approved by the local ethics board (Insubria Board of Ethics, approval number 0033025/2015). Body donors provided written informed consent.

## Conflict of Interest Statement

The authors have no conflicts of interest to declare.

## Funding Sources

The Technische Universität Dresden, Germany, provided funding for the publication of the study.

## Author Contributions

Susanne Menzel assisted with the surgical procedure, as well as drafting and revising the article. Iordanis Konstantinidis, Mario Turri-Zanoni, Marco Valentini, and Paolo Battaglia performed the surgical procedure as experienced ENT surgeons, as well as critical revision of the article. Further, Alberto Macchi, Paolo Giocondo Maria Castelnuovo, and Thomas Hummel contributed to the study conception and critical revision of the article. Giorgio Sileo and Giulia Monti assisted with the surgical procedure and supported it by the critical revising of the article. All authors have approved the final version of this article.

## Data Availability Statement

All data generated or analyzed during this study are included in this article. Further inquiries can be directed to the corresponding author.

## Supplementary Material

Supplementary data

Supplementary data

## Figures and Tables

**Table 1:** Overview of possible positions of an olfactory implant to stimulate the OB

Approach	Procedure	Electrode position	Patient's risk	Degree of difficulty for an experienced surgeon	Electrode placement
1	Endoscopic	Intranasal	Low	Low	Far from OB
2	Endoscopic	Intracranial	Medium	Medium	Difficult to place close to OB
3	Endoscopic	Intracranial	Medium	Medium	Good
4	Endoscopic	Intracranial	Medium	Medium	Good
5	Endoscopic	Intracranial	Relatively high	High	Good
6	Combined	Intracranial	Relatively high	High	Good
