# Supplementary material for: Surgical Approaches for Possible Positions of an Olfactory Implant to Stimulate the Olfactory Bulb
Source: ORL J Otorhinolaryngol Relat Spec. 2023 Mar 30;85(5):253–63. doi: 10.1159/000529563 (PMC10627492; doi:10.1159/000529563)
Supplement: Supplementary file 1 — Supplementary data [file orl-0085-0253-s01.docx]

**Supplementary Legend**

Video 1. Endoscopic intranasal positioning of the electrode.
